# Evolution and interrelation of physical frailty and mild cognitive impairment up to two years after kidney transplantation: a multi-center prospective cohort study

**DOI:** 10.3389/ti.2026.16175

**Published:** 2026-09-21

**Authors:** Nadine Beerli, Manuel Wehrle, Isabelle Binet, Michael Dickenmann, Déla Golshayan, Karine Hadaya, Uyen Huynh-Do, Reto W. Kressig, Sabina De Geest, Oliver Mauthner

**Affiliations:** 1 Institute of Nursing Science, University of Basel, Basel, Switzerland; 2 University Department of Geriatric Medicine Felix Platter, Basel, Switzerland; 3 Clinic of Nephrology and Transplantation Medicine, Cantonal Hospital St Gallen, St Gallen, Switzerland; 4 Department for Transplantation-Immunology and Nephrology, University Hospital Basel, Basel, Switzerland; 5 Transplantation Centre and Transplantation Immunopathology Laboratory, University Hospital Lausanne, Lausanne, Switzerland; 6 Department of Nephrology, University Hospital Geneva, Geneva, Switzerland; 7 University Clinic for Nephrology and Hypertension, University Hospital Bern, Bern, Switzerland; 8 Academic Centre for Nursing and Midwifery, Department of Public Health and Primary Care, KU Leuven, Leuven, Belgium

**Keywords:** chronic kidney disease, end stage renal disease, frailty, kidney transplantation, mild cognitive impairment

## Abstract

Kidney transplantation (KT) is the preferred treatment for kidney failure, providing superior survival and quality of life compared with haemodialysis. However, the demand for transplantation exceeds organ availability, underscoring the need to optimize pre- and post-transplant care. Physical frailty and mild cognitive impairment (MCI) are important predictors of post-transplant outcomes, yet their long-term trajectories remain insufficiently understood. This prospective multicentre longitudinal study, nested within the Swiss Transplant Cohort Study, included 230 adult KT recipients followed for up to 2 years after transplantation. Physical frailty was assessed using the adapted Fried Frailty Phenotype and MCI using the Montreal Cognitive Assessment. Physical frailty declined from 8.3% before transplantation to below 2% during follow-up, while pre-frailty decreased from 54.3% to 27.6% at 2 years. MCI prevalence declined from 42.6% to 31.3%. Longitudinal analyses demonstrated that physical frailty was more dynamic than cognitive function. Better cognitive function at earlier time points predicted lower subsequent frailty, whereas frailty did not significantly predict later cognitive function. These findings suggest that cognitive assessment may help identify patients at risk of persistent frailty and support the routine assessment of both physical frailty and cognitive function before and after KT to improve risk stratification and guide individualized patient care.

## Graphical Abstract

**Figure:**
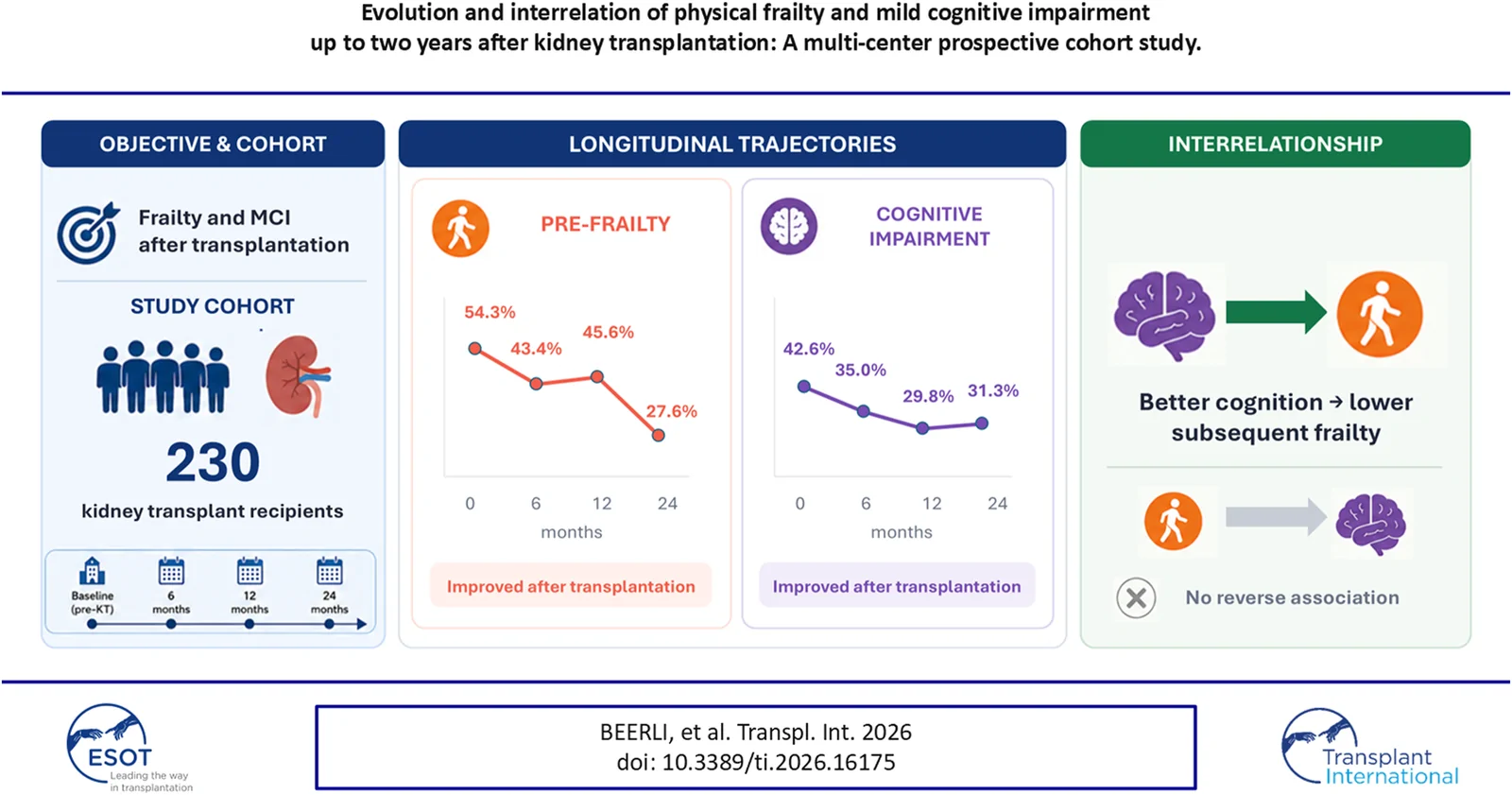

## Introduction

Population ageing and an increasing incidence of chronic disease have led to a growing number of patients with kidney failure being considered for kidney transplantation (KT) [1, 2]. KT is recognised as the most effective treatment option for kidney failure patients and offers significant advantages over haemodialysis treatment in terms of mortality, graft function and quality of life [3–5]. As a result, the demand for grafts is steadily increasing worldwide while the number of available donor organs has been stagnating [6, 7]. Consequently, the demand for kidney transplants considerably exceeds the number of available grafts, which underlines the importance of optimising pre- and post-KT procedures and outcomes. Age significantly impacts transplant results, were individuals of similar age may present with different physical and cognitive conditions. Thus, chronological age (counted in years) alone is an inaccurate representation of patients' functional abilities [8, 9]. Physical frailty, a better predictor of biological age, and MCI were both found to be strong independent predictors of adverse health outcomes and functional decline in ESRD as well as KT recipients [5, 7, 9]. Nevertheless, there is limited evidence on how these two conditions develop long term and how they interact after KT. Therefore, studies that investigate the prevalence, the evolution, and the interrelation of both conditions over time represent an important area of research to contribute informing clinical and policy decision making.

Physical frailty is defined as a state of vulnerability and a decline in functioning across multiple physiological body systems [10, 11]. The Fried Frailty phenotype (FFP) offers the most common way for determining physical frailty in transplantation [12]. This phenotype is based on the five components weakness, slowed walking speed, low level of physical activity, exhaustion, and chronic undernutrition [10, 11]. Depending on the presence of these components, a patient is classified as non-frail; pre-frail; or frail. Although the FFP was initially designed to identify older adults at increased risk for adverse health outcomes, it is presently utilized as a predictive tool for such detrimental health effects across diverse patient populations [10, 13]. For improving its predictive validity, the FFP components have been adapted for KT patients (e.g., chronic undernutrition is measured by asking for loss of appetite instead of asking for weight loss) [14, 15].

In addition to physical frailty, MCI has been shown to significantly influence KT outcomes; MCI is characterized as a syndrome involving cognitive decline that exceeds normative expectations for an individual’s age and educational background, yet does not substantially impair activities of daily living [12, 16]. Physical frailty and MCI are common in patients with chronic kidney disease (CKD) [17–22]. Studies have demonstrated that up to 78% of patients with kidney failure and as many as 58% of KT candidates at the time of transplantation exhibit signs of frailty [18, 19, 22–25]. Both conditions are strong and independent predictors of adverse health outcomes such as postoperative complications, increased rate of early hospital readmission or mortality in chronically ill adults of all ages [22, 26–30]. A study on changes in frailty after kidney transplantation revealed that the prevalence of physical frailty initially increased from 20% immediately after transplantation to 33% 1 month post-transplantation, but subsequently decreased to 27% at 2 months and 18% at 3 months. The findings indicate that physical frailty in kidney transplantation patients is not a stagnant condition, but rather a dynamic physiological state [31].

MCI has in parallel been found to be a strong and independent predictor of increased rates of hospitalization and mortality in different populations [20, 21, 26, 30, 32]. It also adversely affects patients’ decision-making, the adoption of healthy lifestyle behaviours and adherence to medication regimens and can in turn negatively influence health outcomes in the KT population [20, 27, 33]. The prevalence of MCI in CKD patients is high, with figures up to 75%. Therefore, several studies suggest regular screening for MCI in the CKD population to allow early detection [20, 21, 26, 30, 34]. Moreover, physical frailty and mild cognitive impairment appear to contribute to a cycle of physical and cognitive decline and reduced quality of life [20, 35]. Dysregulation of multiple biological and stress-response systems has been implicated in the development of both frailty and cognitive impairment. The coexistence of these conditions may further increase vulnerability and accelerate functional decline [36, 37]. Additionally, physical frailty has been associated with lower cognitive function and poorer cognitive outcomes following the initiation of haemodialysis and after KT [24, 38, 39]. However, despite increasing recognition of the clinical importance of both conditions, little is known about their longitudinal evolution after kidney transplantation or the directionality of their relationship. Understanding whether frailty and cognitive impairment improve, worsen, or influence one another over time may provide important insights into patient recovery following transplantation and may help identify potential targets for intervention. Consequently, the identification and management of frailty and cognitive impairment have become increasingly important in kidney transplantation [19, 20]. While the associations between frailty, MCI, and adverse transplant outcomes have been well documented, substantially less is known about how these conditions evolve following transplantation and whether they influence one another over time. A better understanding of their longitudinal trajectories and interrelationship may help identify mechanisms underlying post-transplant recovery and inform the development of targeted interventions aimed at improving patient outcomes.

The objective of this study was to assess the prevalence of physical frailty and MCI among adult KT recipients at the time of transplantation and to describe their longitudinal evolution and interrelationship during the first 2 years following KT. Given the established associations of both conditions with adverse transplant outcomes, a better understanding of their trajectories may support risk stratification, guide the development of targeted interventions, and inform future studies investigating their impact on long-term transplant outcomes.

## Materials and methods

The current study presents results of the multicenter, prospective cohort study GERAS (ExplorinG frailty and mild cognitive impairmEnt in adult kidney tRansplant recipients to enhance risk prediction for biochemicAl, psychosocial, and health cost outcomeS) [40]. GERAS is nested in the Swiss Transplant Cohort Study (STCS), a long-term open prospective cohort study that has enrolled more than 95% of all KT recipients in Switzerland since 2008 [41, 42]. The GERAS study was approved as a multicenter study by the responsible ethics committee (EKNZ: 2015-235). Figure 1 illustrates the study design, data resources and variables of the GERAS Study. The GERAS study protocol has been published elsewhere [40].

**FIGURE 1:**
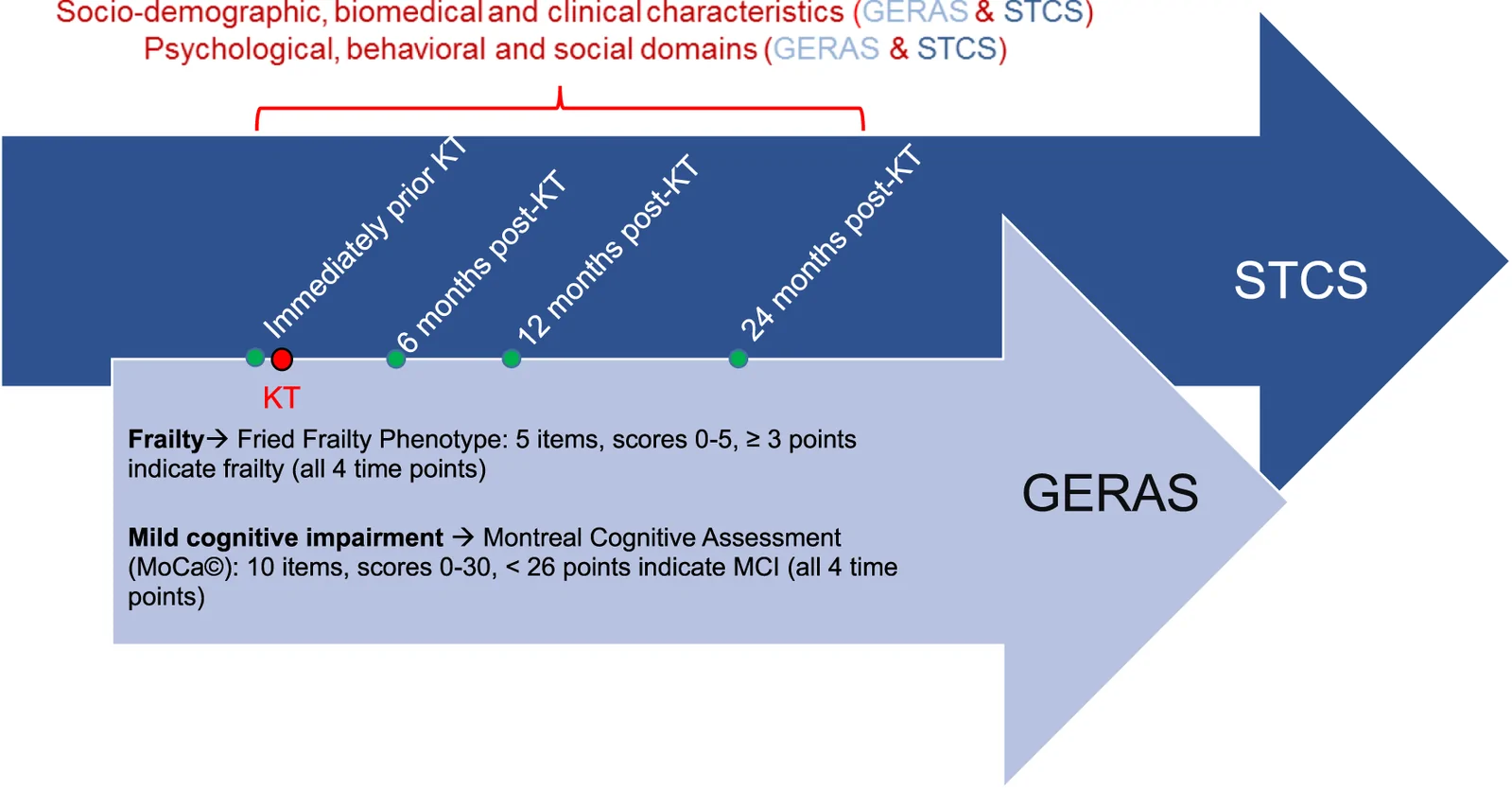
Time frame GERAS study.

### Setting and sample

We included a convenience sample of deceased- and living-donor KT recipients aged ≥20 years who are enrolled in the STCS. Recruitment occurred from March 2016 to July 2019 across four university KT-hospitals and one cantonal KT-hospital in Switzerland. Patients receiving a first or re-implant KT were eligible. We excluded patients that received more than one organ transplant; were not capable to give or understand informed consent; had insufficient knowledge of German, French, English or Italian; or had severe functional impairments (e.g., blindness or wheelchair bound) precluding study assessments. Enrolled participants did not differ based on age and sex from those who were not included in the cohort. With a follow-up time of 2 years, data collection was completed in August 2021.

### Data collection and data management

Potential participants for this study were screened during a routine clinic visit prior to living-donor KT, specifically within a maximum of 3 days before the procedure. For deceased-donor KT recipients, screening occurred upon hospital admission as part of the integrated routine clinical care. Trained data collectors performed informed consent procedures using methods in concordance with the Swiss Ethics regulations. Data collectors were research team members as well as KT center nursing and medical staff. All individuals collecting data were trained in their native language through face-to-face sessions at the respective KT center. Co-investigators of the GERAS project complied with applicable privacy laws, and obtained data were considered confidential with no third-party disclosure. KT recipients were assessed for physical frailty and MCI immediately pre-KT with follow-up evaluations at 6 months, 12 and 24 months post-KT.

To ensure structured and comprehensive patient follow-up, the Secu-Trial™ database (a secure, web-based data platform) had been established. Linking and coding of data from various sources was performed within Secu-Trial™ in collaboration with the STCS data centre and the Clinical Trial Unit, University Hospital Basel [40].

The KT centers received data collection packages including a step-by-step data collection manual [40]. Professional native speakers translated all assessments and questionnaires, i.e., adapted Fried frailty assessment and Montreal Cognitive Assessment (MoCA) from their original language to the three target languages French, German, and Italian [10, 43]. Native speaking members of the research team performed the back translation. Its comparability with the original was discussed in the research team. We pilot-tested the study documents in a convenience sample of four hemodialysis patients, KT candidates and KT recipients from all KT centers to test face validity and estimated time investment. Following the pilot testing we made some adjustments to the wording to improve language clarity.

### Variables and measurements

Physical frailty was assessed by data collectors using the adapted Fried frailty assessment for KT patients [13, 14]. The original five domains of the FFP were developed and psychometrically tested in the Cardiac Health Study [10]. Both, the original and adapted versions have shown good construct and predictive validity in KT cohorts [28, 44–46]. The adapted version requires an approximately 10-min interview by a data collector and evaluates the five domains weakness (handgrip strength, cut-off based on age and sex), slowness (measured habitual walking speed, cut-off based on established measures), low level of physical activity (self-report item on physical activity), lower total energy expenditure (self-report item on subjective exhaustion) and chronic undernutrition (self-report item on loss of appetite) (Table 1) [10, 49]. Every domain is scored as 0 or 1, depending on its absence or presence. We calculated a summary score and grouped patients in non-frail (score 0), pre-frail (score 1–2) or frail (score 3–5) [10].

**TABLE 1:** Fried frailty assessment.

Domain	Procedure	Evaluation
Weakness	Mean values of three tests of maximum grip strength with both hands by the hand-held Jamar dynamometer® [47] were calculated	Weak grip strength: mean value ≤ two standard deviations of sex and age adjusted normative values [48]
Slowness	Time in seconds to complete a 5-m walk, measured, following a standardized protocol	Slow walking speed: Average of three attempts ≥6 s
Low level of physical activity	One closed-ended question: “How often do you engage in activities that require a low or moderate level of energy, such as gardening, cleaning the car or going for a walk?” Answer options: more than once a week/once a week/one to three times a month/rarely	Low level of physical activity: response of “one to three times a month” or “rarely”
Exhaustion	Two closed-ended questions: “In the last week, did you feel on at least 3 days, that everything you did was an effort?” and “In the last week did you feel on at least 3 days, that you could not get going?” Answer options: yes/no.	Exhaustion: response of “yes” to at least one question
Appetite	One closed-ended question: “Have you, in the last 3 months, been eating more/unchanged/less than usual?” Answer options: less/unchanged/more	Chronic undernutrition: response of “less”

Mild cognitive impairment was assessed by data collectors using the Montreal Cognitive Assessment (MoCA) at the time of transplantation [43, 50, 51]. The MoCA is a 10-min assessment recommended for evaluating cognitive function and covers visuospatial and executive functioning, naming, memory, attention, language, abstraction, delayed recall, and orientation. The overall score is 0–30, with a score <26 indicating MCI [43].

Demographic and clinical variables: Chronological age (years), sex (male/female) and pre-KT comorbidities (cancer, endocrine-metabolic or cardiopulmonary disease) were retrieved from the medical patient record and the STCS dataset. Donor type (living donor deceased donor), LOS (days of hospitalisation starting from admission to discharge), hospital readmission rate (count of readmissions within the first 6 months after KT), renal replacement therapy received (none, peritoneal dialysis, haemodialysis), graft loss event post-KT (event, no event), mortality, transplant related complications during hospital stay (yes/no) time on dialysis (years), donor age (age) human leukocyte antigen mismatches (count in numbers) and graft rejection within the first 6 months post-KT (yes/no) were retrieved from the STCS data source and reviewed in the medical patient record.

### Statistics

All analyses were performed in R 3.5.2 for Windows and SAS 9.4. Descriptive statistics of central tendency and dispersion and graphical methods for data visualization were used to report the prevalence of physical frailty and MCI immediately before KT and describe their post-KT evolution. As a sensitivity analysis to account for missing values, we compared the numbers of observations that were not followed up till 24 months post-KT for both, physical frailty and MCI. Regression analysis were performed applying generalized estimating equations to account for the repeated measures within patients [52, 53].

We first considered frailty as the dependent variable explained by the MoCA score and second, by treating MoCA as the dependent variable, with frailty as the explanatory variable. For each of these analyses, we adjusted for time-varying and -invariant confounding using a propensity score, calculated based on modelling the respective independent variables by a set of baseline covariates (i.e., age, gender, donor type, educational level, number of comorbidities, first vs. re-transplantation and dialysis type) as well as prior values of both MoCA and frailty scores (i.e., until two prior visits). Additionally, time-lagged models were added to predict both outcomes at subsequent follow-up visits [52, 53]. The significance level was set *a priori* to 0.05.

## Results

During the recruitment phase, 563 adults underwent kidney transplantation across the five participating Swiss transplant centers. Of these, 348 were assessed for eligibility and 230 fulfilled the inclusion criteria and were enrolled in the study. The discrepancy between the total number of transplant recipients and those screened was primarily attributable to logistical constraints during transplantation procedures, particularly when multiple transplantations occurred simultaneously. If several transplantations were planned simultaneously, recruitment of all recipients was not always possible. 230 KT recipients across the five participating Swiss transplant centers fulfilled the inclusion criteria and were included in the study (Figure 2). As center-specific differences were not part of the study objectives, participants from all centers were analysed as a single multicenter cohort and results are reported for the pooled sample. Table 2 provides an overview of the sample characteristics. The study participant’s mean age was 53.0 years (SD ± 14.4, range 20–75), 25.2% were aged ≥65 years and 31.7% were female.

**FIGURE 2:**
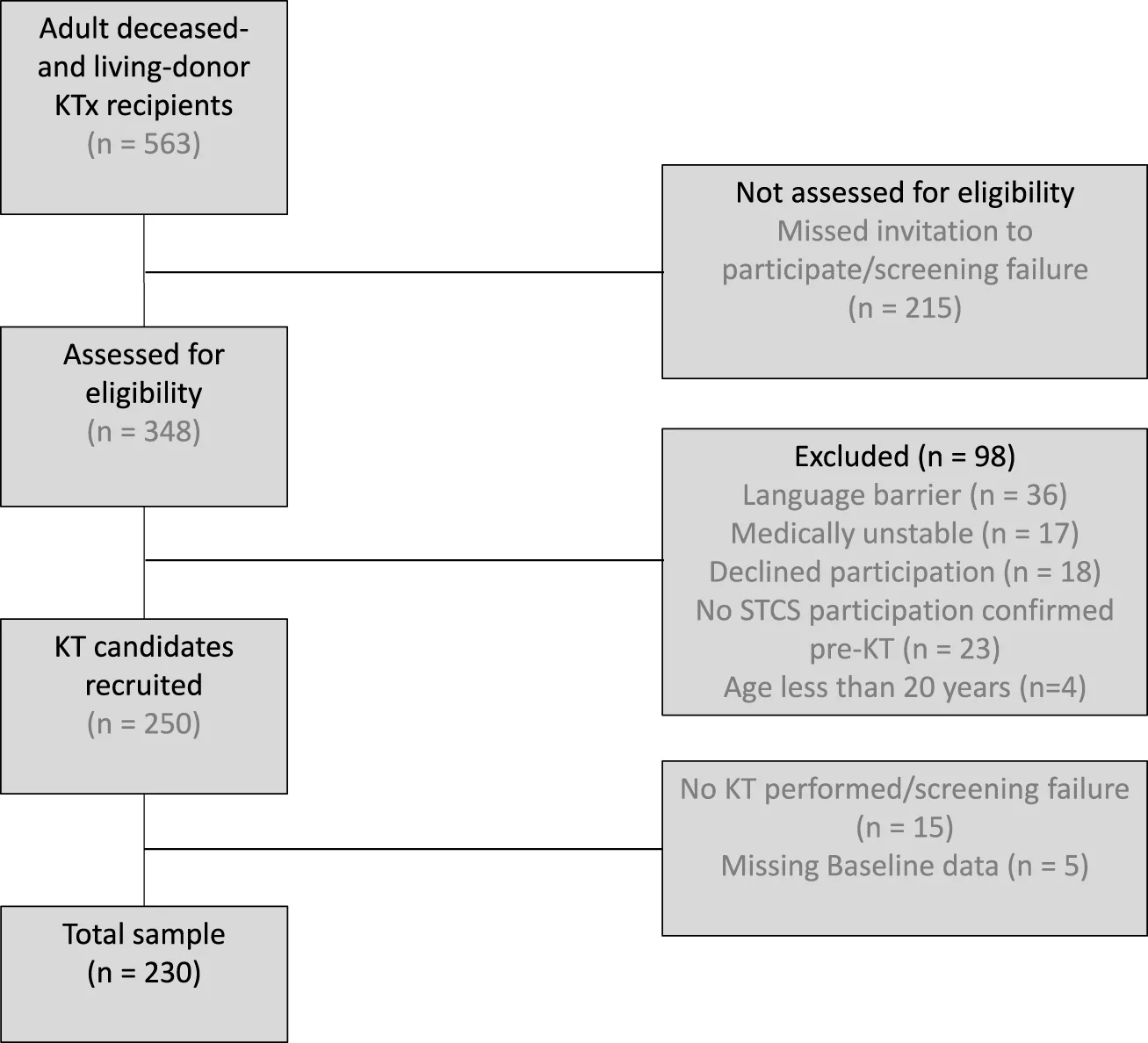
Sample flowchart.

**TABLE 2:** Demographic and clinical variables.

Sample characteristic (n = 230)	Value
Recipient factors Age in years, mean (SD) Age ≥65 years, % Female sex, % Education <13 years, %	53.0 ± 14.4 25.2 31.7 24.3
Comorbidities No comorbidities, % 1–2 comorbidities, % ≥3 comorbidities, %	3.9 39.1 57.0
LOS Days, mean Days, median	13.2 12
Readmission rate No readmission, % Readmission, %	56.5 43.5
Donor type Living % Deceased, %	60.0 40.0
Dialysis type HD, % PD, % None, %	60.4 14.35 24.45
Type of KTx First KTx, % Second KTx, % Third or subsequent KTx, %	85.6 2.2 12.2

SD, Standard deviation; HD, Haemodialysis; PD, Peritonealdialysis; LOS, Length of stay.

### Prevalence of physical frailty and MCI immediately prior to KT

Immediately pre-KT, the prevalence of physical frailty was 8.3%, and 54.3% of the study participants were assessed as pre-frail (Table 3; Figure 3). Physical frailty was determined to be slightly less prevalent in female participants (6.9%) than in male participants (8.9%). Within the age group 65–75 years, the prevalence of physical frailty was lower than in the younger age group 20–64 years (6.9% versus 8.8%.) Overall, the prevalence of MCI was 42.6% (Table 3). The older age group had a higher prevalence than the younger group (67.2% versus 39.0%).

**TABLE 3:** Participants and prevalence of physical frailty and MCI immediately prior to KT.

Participant characteristics	Frailty (n = 230)				MCI (n = 230)		
​	Non-frail (n)	Pre-frail (n)	Frail (n)	Prevalence frail (%)	No MCI (n)	MCI (n)	Prevalence MCI (%)
All participants	86	125	19	8.3%	132	98	42.6%
Age, years 20–64 65–75	63 23	93 31	15 4	8.8% 6.9%	110 21	61 37	35.7% 63.8%
Sex Female Male	29 57	39 86	5 14	6.8% 8.9%	45 87	28 70	38.4% 44.6%
Education level Education <13 years Education ≥13 years	18 68	37 88	1 18	1.8% 10.3%	36 96	20 78	35.7% 44.8%
LOS <13 days ≥13 days	61 25	67 58	7 12	5.2% 12.6%	86 46	49 49	36.3% 51.6%
Dialysis type HD PD None	56 15 14	71 16 38	12 3 4	8.6% 8.8% 7.1%	75 22 34	64 11 22	46.0% 33.3% 39.3%

**FIGURE 3:**
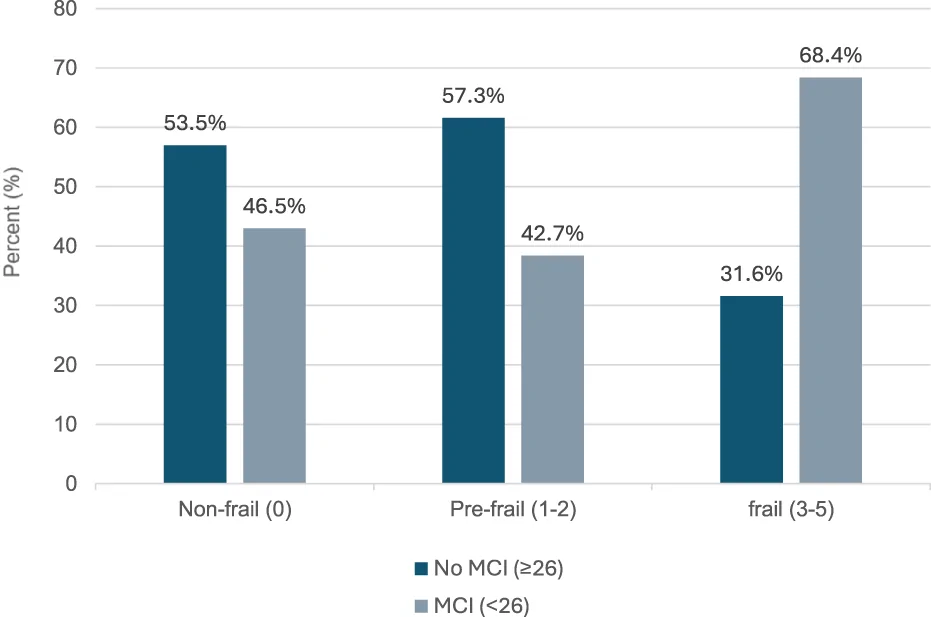
Distribution of physical frailty and MCI immediately pre-KT.

### Evolution of physical frailty and MCI status up to two years post-KT

The prevalence of physical frailty declined over the 2 years follow up period and ranged between 1 and 2% between the 6, 12 and 24 months follow up time points. Physical pre frailty declined initially to 43.4% at 6 months follow up and remained stable at 12 months post KT (45.6%). At the last follow up time point a decrease to 27.6% was detected (Figure 4).

**FIGURE 4:**
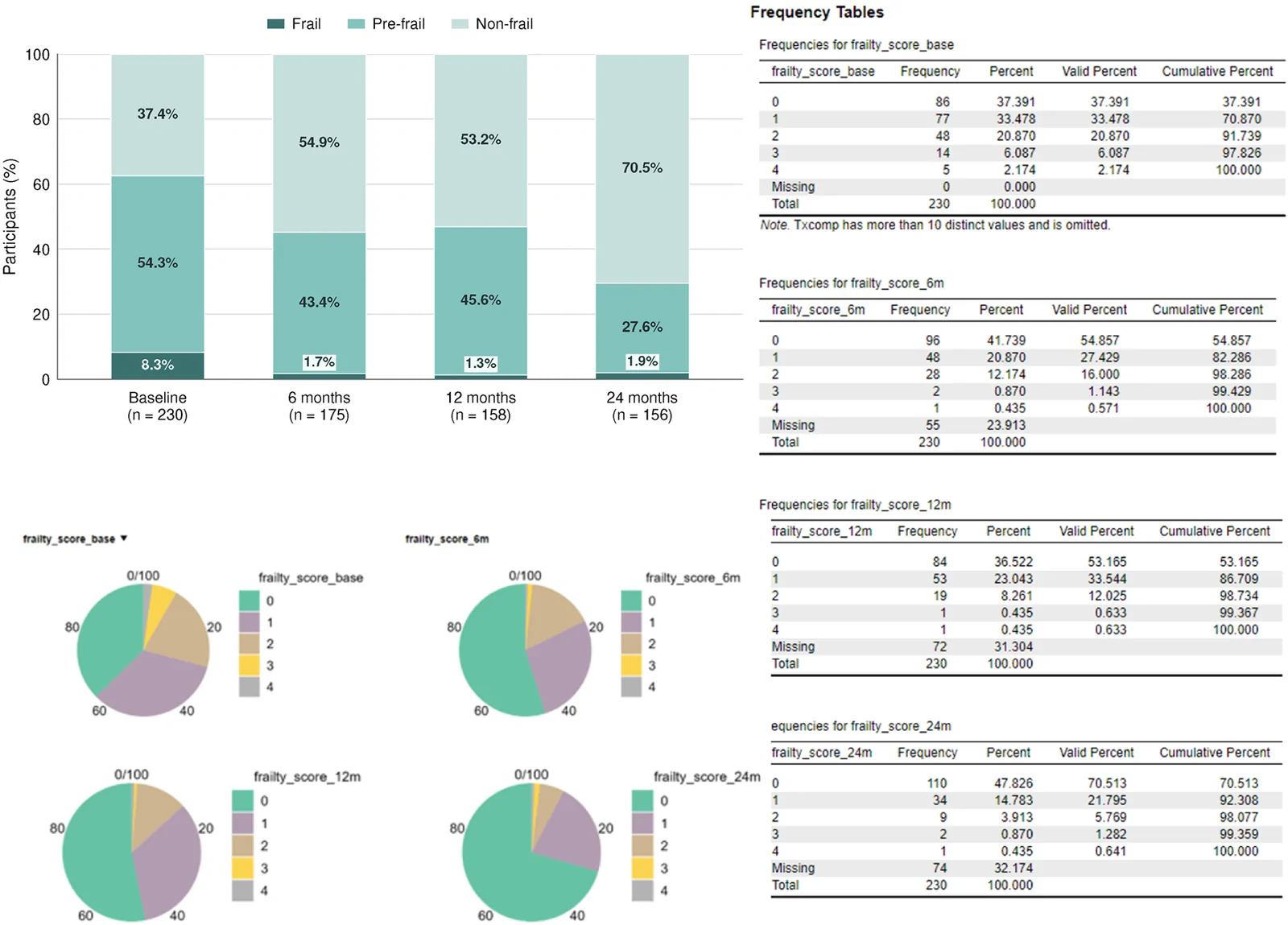
Evolution of physical frailty up to 24 months post KT.

The prevalence of MCI declined to 35.0% at 6 moths, 29.8% at 12 months and 31.3% at 24 months post-KT. Figure 5 presents the evolution of MCI as well as the missing data at each time point.

**FIGURE 5:**
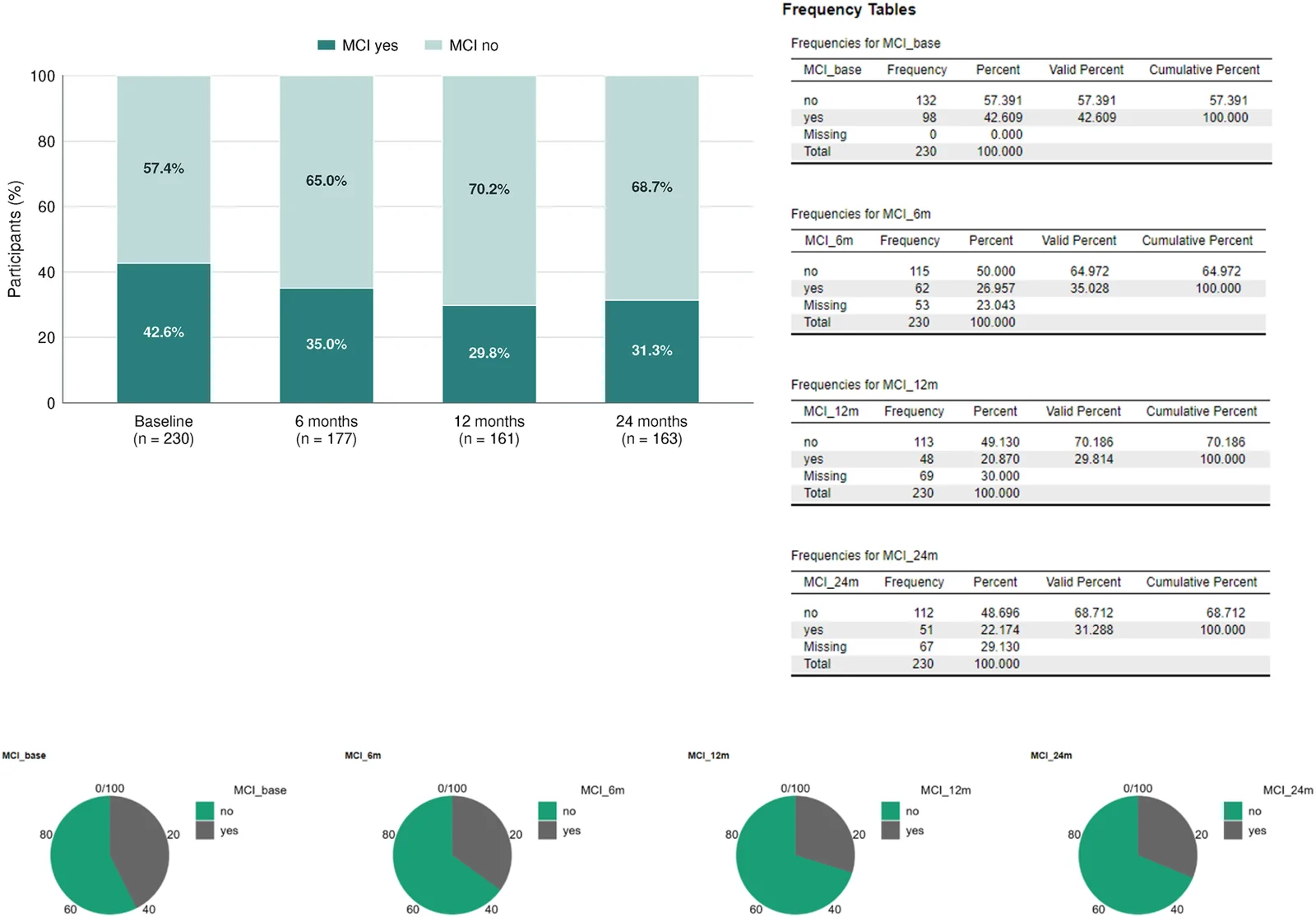
Evolution of MCI up to 24 months post KT.

When calculating the number of observations that did not reach the 24-month post-KT time point, we found that 74 observations (32.2%) for physical frailty and 67 observations (29.1%) for MCI were missing (Supplementary Table).

### The relationship between frailty and cognitive function

The regression analyses revealed a significant association between cognitive function and frailty. The MoCA score was a significant predictor of frailty (OR = 0.92, 95% CI: 0.88–0.96, p = 0.0004) indicating that higher cognitive function was associated with lower frailty levels. Interaction effects between MoCA and time were not statistically significant, suggesting that this relationship remained stable across follow-up visits, thus were omitted from the models (Table 4).

**TABLE 4:** Results of the ordinal logistic regression models.

Model	Odds ratio (95% confidence intervals)	P-value
Frailty scores predicted by MoCA scores	​	​
Concurrent	0.92 (0.88–0.96)	0.0004
Forward lag (one assessment)	0.88 (0.82–0.94)	0.0003
Forward lag (two assessments)	0.97 (0.87–1.08)	0.53
MoCA scores predicted by frailty scores	​	​
Concurrent	0.77 (0.61–0.96)	0.02
Forward lag (one assessment)	0.84 (0.59–1.21)	0.35

Models are adjusted for time, the propensity score and the propensity score squared.

In the time-lagged models, higher MoCA scores at an earlier time point predicted lower frailty at later visits (OR = 0.88, 95% CI: 0.82–0.94; p = 0.0003). The same was not true for frailty being predictor of cognitive function in the future (OR = 0.84, 95% CI: 0.59–1.21; p = 0.35) suggesting that cognitive function is able to predict frailty progression, in an unidirectional way (Table 4).

To assess potential attrition bias, baseline characteristics were compared between participants with and without available 24-month follow-up assessments. Participants without a 24-month MoCA assessment had lower baseline MoCA scores (p = 0.019) and a higher comorbidity burden (p = 0.021), whereas participants without a 24-month frailty assessment had higher baseline frailty scores (p = 0.032).

## Discussion

The objective of this study was to evaluate the prevalence of physical frailty and MCI among adult KT recipients at the time of transplantation and to describe their evolution up to 2 years post-KT. We found that in our study population, the prevalence of physical frailty amounted to 8.3% and that of MCI to 42.6%.

The prevalence of physical frailty in KT patients in our study was lower than that reported in the literature (8% compared to up to 20%) [6, 8, 9, 18, 44, 54]. In contrast, the percentage of patients with physical pre-frail status was higher than values previously reported in an international study (54% compared to 33%) [44, 45]. This finding might result from the fact that healthcare systems differ among countries, making direct comparisons difficult. Nevertheless, the average time on the waitlist for KT in Switzerland was 2.6 years in 2018, which is low compared to other European countries and the US (Swisstransplant, ERA-EDTA, UNOS). A shorter waiting time has been shown to be beneficial in various studies, whereas a prolonged time on the waitlist has been associated with negative outcomes after KT [3, 55]. The relatively short waiting time may be one factor explaining why many patients were only in the physical pre-frail stage and not yet further advanced. Interestingly, pre-frailty was more prevalent among pre-emptive transplant recipients than among patients receiving haemodialysis. This difference was not explained by age in our cohort and may instead reflect other patient characteristics or selection factors that were not investigated in the present study. A further notable finding was the lower prevalence of physical frailty among recipients aged 65–75 years compared with those aged 20–64 years. Although this observation appears counterintuitive, it is likely explained by the selection process for kidney transplantation. Older transplant recipients represent a highly selected population who have undergone extensive medical and functional evaluation and are therefore more likely to have preserved physiological reserve than older individuals with kidney failure in the general population [6]. In contrast, younger recipients may accumulate a substantial burden of chronic disease, dialysis-related complications, and multimorbidity that contribute to frailty despite their younger chronological age [56]. This observation further supports the concept that chronological age alone is an insufficient surrogate for biological age in kidney transplantation and reinforces the importance of incorporating frailty assessment into transplant evaluation irrespective of age [3, 6, 12, 54]. Nevertheless, given the relatively small number of frail participants in our cohort, this finding should be interpreted with caution and warrants confirmation in larger prospective studies. Similarly, although physical frailty appeared to be more prevalent among participants with a higher educational background, this finding should be interpreted cautiously, as participants with higher educational attainment were proportionally overrepresented in our cohort and this imbalance may have influenced the observed distribution.

This study revealed a high prevalence of MCI among kidney transplant recipients immediately prior to transplantation, consistent with previous reports [20, 36, 57, 58]. Although pre-transplant anxiety related to the anticipated transplantation procedure may have influenced cognitive performance in some individuals, baseline assessments were conducted under standardized and calm clinical conditions as part of routine pre-transplant evaluations. The observed reduction in MCI prevalence following transplantation aligns with longitudinal studies demonstrating improvements in cognitive function after kidney transplantation, particularly during the first postoperative year, although residual cognitive impairment remains common [20, 36]. Therefore, the observed trajectory is more likely to reflect genuine post-transplant cognitive recovery than a transient effect of perioperative stress. Evidence regarding longer-term cognitive trajectories is less consistent, with some studies reporting persistent impairment or late cognitive decline, whereas others demonstrate sustained improvement or stabilization [20, 57]. Consistent with recent longitudinal evidence, we observed a decline in MCI prevalence from 42.6% before transplantation to 29.8% at 12 months, followed by stable prevalence at 24 months. These findings suggest that cognitive impairment after KT is not necessarily progressive but rather reflects an early phase of cognitive recovery followed by relative stability over the first two years after transplantation. Despite this favourable trajectory, persistent cognitive impairment remained common, highlighting the importance of integrating cognitive screening and tailored support into routine post-transplant care to optimize self-management, medication adherence, and long-term clinical outcomes [20]. Although frailty improved and cognitive function remained relatively stable over time, these findings should be interpreted in light of the observed attrition. Participants with poorer baseline cognitive function and greater physical frailty were more likely to have missing 24-month follow-up assessments, which may have resulted in a modest overestimation of the observed longitudinal improvements.

Post-KT, the study observed a marked decline in the prevalence of physical frailty, decreasing to between 1% and 2% at the six, twelve, and twenty-four-month follow-ups. Similarly, pre-frailty levels decreased significantly, from 43.4% at six months to 27.6% at twenty-four months. This trend aligns with existing literature suggesting that rehabilitation and supportive care post-transplant can lead to improved physical outcomes. Studies have demonstrated that structured rehabilitation programs can improve physical functioning in kidney transplant recipients. Recent studies further indicate that structured exercise-based prehabilitation can improve exercise capacity, muscle strength, physical performance, and muscle morphology in kidney transplant candidates, including those with frailty. These findings support the integration of individualized exercise interventions into multidisciplinary care across the kidney transplantation pathway [59, 60]. In terms of cognitive health, MCI prevalence also decreased over time, dropping from 35.0% at six months to 31.3% at twenty-four months. This reduction, although less pronounced than that observed in physical frailty, indicates that cognitive function can improve post-transplant, possibly due to enhanced physical health and psychosocial factors.

Regression analyses further revealed a significant association between cognitive function and frailty, suggesting that cognitive decline precedes subsequent physical resilience. This is important in identifying patients at risk. Moreover, if this would constitute a causal pathway, then physical frailty may respond to interventions targeting cognitive function, thus early cognitive assessments in kidney transplant patients may help mitigate frailty progression over time.

The strength of this study lies in its prospective, longitudinal design, which encompassed a 24-month follow-up period post-KT and employed a multicentric approach. However, two significant limitations of this study are the reliance on a convenience sample, which led to a considerable number of missed patients, and the presence of missing data at several follow-up time points particularly in later visits, reduced the statistical power to detect weaker associations and may have introduced bias, which could not be addressed by the use of less missingness-sensitive random-effects models (i.e., because of convergence problems). The follow-up duration may also have been insufficient to capture the long-term impact of frailty on cognitive decline. Further, the potential attrition bias should be considered when interpreting the longitudinal findings, although the mixed-effects models included all available observations and were therefore able to accommodate intermittent missing follow-up data. Future studies should aim to extend the follow-up period and incorporate more detailed assessments of both cognitive and physical function, such as domain-specific neuropsychological tests and further objective frailty measures. Moreover, interventional studies investigating whether improving cognitive function can mitigate frailty progression in kidney transplant recipients would be valuable.

In conclusion, this study underscores the dynamic nature of physical frailty in kidney transplant recipients, demonstrating significant improvements over time, while MCI remained more stable. These findings highlight the critical importance of ongoing monitoring of both physical frailty and MCI in clinical settings. This study further provides evidence that cognitive function plays a significant role in frailty progression among kidney transplant patients, whereas frailty does not appear to substantially impact cognitive decline within the observed timeframe. These findings highlight the importance of early cognitive screening and intervention strategies to improve long-term outcomes in this patient population. Healthcare providers should implement routine assessments of frailty and cognitive function to identify at-risk patients early to adapt how healthcare services are being provided. Additionally, developing targeted interventions aimed at reducing frailty and improving cognitive health is essential to enhance patient outcomes and overall quality of life post-KT. By prioritizing these aspects of patient care, clinicians can better support the long-term wellbeing of kidney transplant recipients.

## Data Availability

The raw data supporting the conclusions of this article will be made available by the authors, without undue reservation.
