# Supplementary material for: Evolution and interrelation of physical frailty and mild cognitive impairment up to two years after kidney transplantation: a multi-center prospective cohort study
Source: Transpl Int. 2026 Sep 21;39:16175. doi: 10.3389/ti.2026.16175 (PMC13635986; doi:10.3389/ti.2026.16175)
Supplement: Supplementary file 1 [file Table1.docx]

**Supplementary Table: Comparison of baseline characteristics between participants with and without available 24-month follow-up assessments for cognitive function (MoCA) and physical frailty (FFP)**

| **Characteristic** | **MoCA available (n = 162)** | **MoCA missing (n = 68)** | **p-value** | **FFP available (n = 156)** | **FFP missing (n = 74)** | **p-value** |
| --- | --- | --- | --- | --- | --- | --- |
| **Age, years** | 51.90 ± 14.14 | 56.18 ± 11.92 | 0.053 | **51.62 ± 14.20** | **56.43 ± 11.81** | **0.026** |
| **Baseline MoCA score** | **25.59 ± 3.43** | **24.53 ± 3.43** | **0.019** | **25.55 ± 3.51** | **24.69 ± 3.29** | **0.029** |
| **Baseline frailty score** | **0.97 ± 0.99** | **1.09 ± 1.07** | 0.322 | **0.92 ± 0.97** | **1.23 ± 1.07** | **0.032** |
| **Comorbidity count** | **2.66 ± 1.41** | **3.00 ± 1.69** | **0.021** | **2.78 ± 1.45** | **3.14 ± 1.80** | 0.219 |

**Abbreviations:** FFP, Fried Frailty Phenotype; MoCA, Montreal Cognitive Assessment; SD, standard deviation.

**Note:** Data are presented as mean ± SD. Continuous variables were compared using the Mann–Whitney U test because the variables were not normally distributed.
